# Challenges related to human papillomavirus (HPV) vaccine uptake in Minnesota: clinician and stakeholder perspectives

**DOI:** 10.1007/s10552-021-01459-5

**Published:** 2021-07-10

**Authors:** Nicholas Yared, Molly Malone, Estee Welo, Inari Mohammed, Emily Groene, Matthew Flory, Nicole E. Basta, Keith J. Horvath, Shalini Kulasingam

**Affiliations:** 1 Division of Infectious Disease, Department of Medicine, Henry Ford Health System, 2799 West Grand Boulevard, Detroit, MI 48202 USA; 2 Division of Epidemiology and Community Health, School of Public Health, University of Minnesota, Minneapolis, MN USA; 3 Minnesota Cancer Alliance, American Cancer Society, Saint Paul, MN USA; 4 Department of Epidemiology, and Occupational Health, McGill University, Montreal, QC Canada; 5 Department of Psychology, San Diego State University, San Diego, CA USA

**Keywords:** Human papillomavirus, Vaccines, Cancer prevention, Social media

## Abstract

**Background:**

Human papillomavirus (HPV) vaccination rates among adolescents are increasing in Minnesota (MN) but remain below the Healthy People 2020 goal of 80% completion of the series. The goal of this study was to identify messaging and interventions impacting HPV vaccine uptake in MN through interviews with clinicians and key stakeholders.

**Methods:**

We conducted semi-structured key participant interviews with providers and stakeholders involved in HPV vaccination efforts in MN between 2018 and 2019. Provider interview questions focused on messaging around the HPV vaccine and clinic-based strategies to impact HPV vaccine uptake. Stakeholder interview questions focused on barriers and facilitators at the organizational or state level, as well as initiatives and collaborations to increase HPV vaccination. Responses to interviews were recorded and transcribed. Thematic content analysis was used to identify themes from interviews.

**Results:**

14 clinicians and 13 stakeholders were interviewed. Identified themes were grouped into 2 major categories that dealt with messaging around the HPV vaccine, direct patient–clinician interactions and external messaging, and a third thematic category involving healthcare system-related factors and interventions. The messaging strategy identified as most useful was promoting the HPV vaccine for cancer prevention. The need for stakeholders to prioritize HPV vaccination uptake was identified as a key factor to increasing HPV vaccination rates. Multiple providers and stakeholders identified misinformation spread through social media as a barrier to HPV vaccine uptake.

**Conclusion:**

Emphasizing the HPV vaccine’s cancer prevention benefits and prioritizing it among healthcare stakeholders were the most consistently cited strategies for promoting HPV vaccine uptake. Methods to combat the negative influence of misinformation about HPV vaccines in social media are an urgent priority.

## Introduction

The HPV vaccine is recommended for administration to all adolescents in the USA for the prevention of HPV-related cancers and anogenital warts [1]. Uptake of the HPV vaccine in the USA has been lower than that of other adolescent vaccines and remains below the Healthy People 2020 goal of 80% completion of the vaccine series [2]. HPV vaccine uptake rates also vary regionally and by state. In Minnesota, data from the Minnesota Department of Health (MDH) indicate that the rate of uptake of the HPV vaccine among adolescents aged 13 to 17 in 2018 was 76.7% for one dose and 58.1% for completion of the series [3]. While these uptake rates compared favorably to the USA as a whole (68.1% for receipt of at least one dose and 51.1% for completion of the series), they remain below the Healthy People 2020 goal [3].

Clinician recommendations influence parents’ opinion of their adolescents’ receipt of the HPV vaccine [4, 5]. Multiple studies have linked a clinician’s recommendation to vaccinate adolescents for HPV to greater willingness to follow through with vaccination. Clinicians play an important role in ensuring that adolescents receive the HPV vaccine [4, 5]. Given their importance in discussing the HPV vaccine with adolescents and their parents, prior efforts have been made to assess clinicians’ opinions about prioritizing HPV vaccine delivery, structuring care teams to optimize vaccination efforts, and addressing barriers to vaccination [6]. There are also ongoing efforts to evaluate educational interventions targeted at clinicians to promote HPV vaccination [7]. Key stakeholders involved in HPV vaccination, such as those responsible for quality improvement initiatives at health maintenance organizations or affiliated with local and state health departments, can also play a role in supporting HPV vaccination.

In order to better understand what methods to employ to improve HPV vaccine uptake, we aimed to first understand what messages were perceived by clinicians and healthcare stakeholders that were thought to impact uptake of the HPV vaccine. Additionally, we aimed to gather further knowledge of best practices and innovative strategies being used to promote HPV vaccine uptake that would provide information to design future interventions. We performed a thematic content analysis of interviews conducted with practicing clinicians and HPV-focused stakeholders in Minnesota to address these aims.

## Materials and methods

We conducted semi-structured key participant interviews with 14 clinicians and 13 key stakeholders in Minnesota regarding HPV vaccination. The interviews took place from July 2018 to August 2019. Clinicians were interviewed by an infectious disease fellow physician and PhD student in epidemiology. Stakeholder interviews were conducted by an Epidemiology PhD and the project’s principal investigator as well as a PhD student in epidemiology. We asked about effective messaging strategies, quality improvement initiatives, and support for HPV vaccine-related legislation in Minnesota. This research was considered exempt by the University of Minnesota Institutional Review Board (IRB).

Key informant interviews were structured according to a guide comprised of questions asked of each clinician or stakeholder. Clinician and stakeholder interview questions were developed by a team consisting of an epidemiologist with experience involving development of HPV screening guidelines, other epidemiologists with experience conducting content analysis, and a practicing infectious disease fellow physician. All interviews were audio recorded and transcribed verbatim by study personnel.

Thematic content analysis was used to analyze the data. The framework for content analysis was to identify key phrases involving messaging around the HPV vaccine and strategies to improve uptake among clinicians and healthcare-related stakeholders. Prior to conducting interviews, the coding scheme for clinician interviews was developed by an infectious disease fellow physician and MPH student based around pre-determined interview questions. The coding scheme for stakeholder interviews was developed by the project’s principal investigator.

The content of the transcribed interviews was independently reviewed by two researchers. Each reviewer first independently read through the entire text of each transcribed interview once and identified key words or phrases associated with barriers or facilitators to HPV vaccination. Subsequently, the key words and phrases that were identified were compared between the two researchers via a second read-through of each interview with patterns among the coded words used to identify specific themes. Identified themes were reviewed in research team sessions to further refine them and lessen the risk of individual reader bias.

### Clinicians

A target sample size of fifteen individuals was set prior to clinician recruitment. Clinicians were included if they were physicians or nurse practitioners currently working in an ambulatory clinic in Minnesota and were involved in discussions about the HPV vaccine with their patients. Additionally, only clinicians who were able to order and prescribe the HPV vaccine were eligible to participate. Retired clinicians and those not actively engaged in an ambulatory primary care practice were excluded. Affiliates at the Minnesota chapter of the American Cancer Society and the Minnesota Department of Health aided in identifying clinicians for this study through purposive sampling. Outreach to clinicians occurred via e-mail contact by a research assistant. Potential participants were e-mailed the interview questions and a consent form prior to the interview. Consent to participate was confirmed via an e-mail reply message or verbally prior to the start of the interview.

Participants were asked a series of open-ended questions related to their communication about the HPV vaccine as shown in Table 1. Topics that were addressed included concerns related to receipt of the HPV vaccine, differences in messaging strategies used for different sexes and population subgroups, and quality improvement projects aimed at increasing uptake of the HPV vaccine within each clinician’s specific healthcare setting.

**Table 1:** Clinician interview questions

1. What are the most common questions or concerns that you have received from patients and their parents about the HPV vaccine? Which concern is the most challenging to address in your opinion?
2. When you encounter concerns about the HPV vaccine in clinic? How do you respond?
3. How often do time pressures during clinic visits impact your ability to discuss the HPV vaccine with patients and their parents/guardians?
4. What strategies have you or your clinic used to ensure that patients return for additional doses of the HPV vaccine in a timely manner?
5. Are there any differences in messages that you use to promote the HPV vaccine for boys vs. girls?
6. Are there any specific populations in your clinic to whom you tailor your messages about the HPV vaccine in a specific way? If so, please specify how your messaging differs for these groups
7. Has your clinic implemented any quality improvement projects aimed at improving uptake of the HPV vaccine? If so, please describe them
8. What do you think is the most effective strategy for increasing the rate of uptake of the HPV vaccine in your clinic?
9. Is there anything else that you would like to tell us about your experience with promoting the HPV vaccine?

### Stakeholders

We also interviewed thirteen key healthcare stakeholders operating in Minnesota who were identified via purposive sampling. Identification of stakeholders was achieved with the assistance of the Minnesota Health Department and the Minnesota Chapter of the American Cancer Society. Outreach to stakeholders occurred in the same manner as was done with clinicians.

Participants were asked a series of open-ended questions adapted from a previously published survey (Table 2) [8]. Questions were asked about facilitators for and barriers to receipt of HPV vaccination in Minnesota, perceptions about HPV vaccination at the organizational level, and HPV vaccination collaborations with stakeholders from across the state. Stakeholders were also asked about possible next steps for legislation to help improve HPV vaccine uptake rates statewide.

**Table 2:** Stakeholder Questions (adapted from Cartmell et al., Papillomavirus Res 2018;5:21–31)

Perceptions about barriers and facilitators to HPV vaccination at the state level
1. What factors in our state facilitate HPV vaccination?
2. What are the barriers to HPV vaccination in our state?
3. You mentioned different barriers to HPV vaccination in our state. Thinking about these barriers, what type of strategy or strategies do you think would be most effective for improving HPV vaccination rates in our state?
4. Are you aware of any vaccination strategies that been used to improve vaccination rates in specific subgroups within the state of Minnesota? By specific subgroups we mean rural populations, immigrants, specific racial or ethnic groups, LGBTQ youth or young adults or boys only
5. When you think of the health outcomes that an HPV vaccination program is aimed at reducing, what are those health outcomes?
Perceptions about HPV vaccination at the organization level
1. What is your organization’s HPV vaccination coverage rate for the following: Adolescents aged 9 to 10 years? 11 to 14 years? 15 + ?
2. What is the range of HPV vaccination coverage for adolescents among the clinics in your system?
Are you able to determine HPV vaccination rates at the provider level? If yes, do you use this information to work with your providers to improve HPV vaccination rates?
3. To what extent is improving HPV vaccination rates a priority for your organization?
4. Can you tell me about any quality improvement initiatives or strategies that you have in place for HPV vaccination? What are your thoughts about the effectiveness of this/these initiatives in increasing HPV vaccination in your organization?
Public health immunization program
1. What impact do you feel that the statewide immunization registry has on HPV vaccination in our state?
2. How do you feel that the state vaccination program influences HPV vaccination in our state?
3. Have you heard about the Minnesota Department of Health Assessment, Feedback, Incentives, and eXchange (AFIX) or enhanced AFIX? If yes, what impact do you think these programs have on HPV vaccination rates in our state and if applicable on your organization’s HPV vaccination rates?
4. What else about the public health immunization program and HPV vaccination is important for us to know?
HPV vaccination linkages with statewide stakeholders:
1. Tell me about any collaboration that you’ve been involved with to improve HPV vaccination in the state, and briefly assess the effectiveness of the collaboration in accomplishing this goal
2. Who do you think are the key stakeholders who should be included in developing strategies to maximize HPV vaccination rates?
Legislation
1. Do you think we need any legislation to improve vaccination rates in our state and if so, what type of legislation do you think is needed?
2. Do you think any specific legislation is needed for HPV vaccines in particular? If yes, what type of legislation do you think is needed?
3. What types of legislation do you think our state legislators would support?
*Final Question* What else about HPV vaccination is important for us to know?

## Results

A total of 27 semi-structured interviews were completed with 14 clinicians and 13 stakeholders. Upon completion of content analysis, we identified three larger thematic categories addressing messaging and interventions impacting HPV vaccine uptake. These three thematic categories included messaging pertaining to patient–clinician interactions around the HPV vaccine (Table 3), concerns around external messaging sources not involving direct patient–clinician interactions (Table 4), and interventions related to healthcare system-related factors (Table 5).

**Table 3:** Patient–Clinician Themes around HPV Vaccination

Theme	Definition	Representative quote
Lack of knowledge/Awareness of the HPV Vaccine	Text describing parental lack of knowledge or awareness of HPV and the HPV vaccine	*I think there is also… more education that needs to be out there for boys needing the vaccine because I think that is fairly new.* (*Stakeholder 11*)
Recommended age is too young	Text describing parents asking providers if their child is too young to receive the vaccine	*Addressing the fact that you are offering a vaccine prior to sexual activity to young people starting at the age of eleven. That often takes parents aback quite a bit.* (*Clinician 9*)
Sexual Activity	Text describing parental concerns about their child becoming promiscuous or engaging in early sexual activity	*The main concern patients had – one is the way we introduced HPV when we did it – that it is, you know, a vaccine which prevents STIs. Some of them really didn’t want to give it to their children because they perceived it as, you know, it’s going to make them more promiscuous. They are going to become sexually active…* (*Clinician 4*)
Vaccine side effects and safety	Text describing concerns about vaccine safety or side effects (i.e., fainting, pain, etc.)	*I get a lot of questions about side effects, ‘I’ve heard bad things’ or ‘I’ve heard that a child has fainted’ or something like that…* (*Clinician 3*)
Clinician–patient (Parent) communication	Text describing the importance of provider communication to patients and parents	*At the clinic in the provider’s office, providers are still not giving a strong recommendation for HPV vaccine, assuming that the HPV would be a point of controversy with parents. There is very little reassurance, and providers should be making that presumptive recommendation and be more comfortable reassuring parents to vaccinate their children.* (*Stakeholder 5*)
Adolescent opinions	Text describing how adolescent patient opinions factor into whether or not they are vaccinated, addressing adolescent concerns	*You know it’s right after the shot um in terms of kids asking about ‘I’ve heard it really hurts’ I think that you know that’s true of kids anxieties with vaccines in general… we try to emphasize with all shots you know that we’re gonna [sic] make it as comfortable as we possibly can and it is self-limited…* (*Clinician 3*)

**Table 4:** External Messaging Themes around HPV Vaccination

Theme	Definition	Representative quote
Social media and false information	Text describing clinicians explaining encounters with parents who find false information online	*I would say the concern that is the most challenging to address is I have some families who have been on social media [sic] things about side effects as related to the HPV vaccine, specifically, like chronic pain…* (*Clinician 8*)
Anti-vaccine movement	Text describing provider encounters with people who do not support vaccination (of any kind)	*The most challenging is when they have a specific family member, like a mother-in-law or a spouse, who is strongly anti-vax. Where they know they’re gonna [sic] get specific criticism for making a choice counter to that family member…* (*Clinician 2*)
Early age benefit	Text describing how a provider messages the efficacy of receiving the vaccine at a younger age, and how this could enhance immune response	*Why are we having to do this at this young of an age? But, it’s simple to answer – maybe tell them that, you know, studies have shown that the best immunity occurs if you can complete the vaccine in this age group, so…* (*Clinician 9*)
Greater provider education	Text describing a need for more comprehensive education on HPV, HPV vaccination, and the training necessary to facilitate it	*I think the most effective strategy would be doing some provider or clinician education around making that presumptive bundle recommendation… And then I think being able to also provide some training and education on what to do when you get a hesitant parent. You know how to navigate that discussion.* (*Stakeholder 12*)
Personal experience	Text describing a provider using personal experience to recommend the vaccine, oftentimes regarding the vaccination of their own children	*I typically share that my children have gotten or will get it and that it’s our only vaccine that prevents cancer.* (*Clinician 10*)
Strong provider recommendation	Text describing a provider recommending the vaccine at every visit type with strong recommendation language and confidence	*I think having a conversation with the families to make sure they understand why we’re doing it and the importance of it – the clinician-to-family conversation. We also present it as part of the recommended immunization series, but to us, it’s the same … It’s an important vaccine that we highly recommend …* (*Clinician 8*)
Promotion via social media/TV	Text describing the use of social media or television to promote messaging for HPV vaccination	*I’ve seen education through media campaigns (specifically through TV ads on educating parents and kids about the vaccine). The real important thing I’ve seen highlighted is that boys need to be vaccinated. I think there has been a misconception that it should be just for girls, but boys and girls should be vaccinated.* (*Stakeholder 4*)
Normalize the vaccine	Text describing the use of normalizing the HPV vaccine along with other recommended adolescent vaccines	*I think if there’s three vaccines due, you recommend all three vaccines. You don’t say ‘Your child needs the TDAP and the Meningococcal vaccine, and I also want to talk to you about a very special recommendation for a very special vaccine that I really strongly recommend, the HPV vaccine.’ That makes the vaccine sound very weird and different.* (*Clinician 5*)
Parallel healthcare example	Text describing a provider giving a similar example of preventive measures and study results that address the benefits and common concerns	*Well, just like with putting young women on birth control pills for acne, the studies show consistently that in their minds the two are so unrelated that it just doesn’t make a difference. When kids decide to have sex, it’s not because they have protection.* (*Clinician 2*)
Future risk uncertainty	Text describing a provider warning parents that they cannot control for the risk of infection of HPV of their child’s future partner	*I know it’s because they just imagine that they’re raising their daughters that there’s no way in the world they’d ever have pre-marital intercourse, then I’ll often say ‘That’s great, but you have no control over who the person they marry has … if they’ve ever had one partner before.* (*Clinician 2*)
Sex-specific messaging	Text describing tailoring messages based on gender	*Sometimes in parents of boys, they’ve heard that it’s a cervical cancer vaccine, so you have to emphasize that that we’re protecting um the boys from those head and neck cancers…* (*Clinician 3*)
Cancer prevention	Text describing presenting the HPV vaccine as cancer prevention (framing)	*I think the concern about cancer and transmission rings home.* (*Clinician 7*)

**Table 5:** Healthcare system-related themes around HPV vaccination

Theme	Definition	Representative quote
Access	Text describing access and availability of the HPV vaccine	*Having the immunizations available* (*shelf life of vaccine, cost to maintain the inventory*)—*if the vaccine is not available to them* = *missed opportunity. Access to the vaccine.* (*Stakeholder 8*)
Financial incentives	Text describing the use of media to advertise financial incentives to vaccinate for HPV	*We have a member voucher, so we will actually give $100 to members who qualify, who complete the HPV vaccination series.* (*Stakeholder 7*)
Organizational priority to increase vaccine uptake	Text describing a participant’s perception of their organization’s level of priority to increase HPV vaccination uptake	*Well, if I could say on a scale of 1 to 10, it’s not a 0 and it’s not a 10… maybe an 8 because it is publicly reported.* (*Stakeholder 2*)
Reporting of vaccination rates	Text describing the use of reporting a clinic or provider’s vaccination rates as a way to increase uptake	*I think one strategy that is really important that Minnesota has done is the Minnesota Community Measurement – they have added this as a measure for providers to be aware that you are getting scored on against your peers for this particular measure.* (*Stakeholder 4*)
Leveraging the EMR	Text describing electronic medical record (EMR) prompts that remind providers to communicate with patient/parent that they are due to be vaccinated	*Well, we had to work hard to modify EPIC, which is our electronic medical record system, to bring it up to date with the two-dose vaccine. And we’re currently struggling with EPIC to fix the problem of young adults who start the vaccine and then age out of 26. Like the national recommendations we hold the that they who began the series should completed. But EPIC currently doesn’t recommend it for people 27 years or older even for the second and third doses…* (*Clinician 5*)
Maximize administration opportunities	Text describing increasing the number of venues in which the HPV vaccine is offered	*We also have started offering the HPV vaccine in our express clinics. We have two express clinics in the community where … they’re located at supermarkets* (*Clinician 5*)
Clinic workflow and scheduling	Text describing specific process changes within a clinical venue that are undertaken to streamline discussing and administering the vaccine	*If they’re in for any other visit outside of a yearly physical, then the rooming process includes checking the vaccine status for anybody even if it’s not for their annual visit…* (*Clinician 10*)
Utilize minnesota immunization information connection (MIIC) Data	Text describing the use of MIIC data in various clinics or healthcare systems	*Unfortunately, all providers are not using MIIC. Not sure as to what percent are not using it. When we just use MIIC, our vaccination rates are very low compared to when we use Chart Review, they’re much higher. Don’t know of anyone who has published on this. Looking at the health plan data vs the MIIC data gives an idea of how you’re doing.* (*Stakeholder 3*)
Mandate vaccine	Text describing a participant suggesting that the vaccine should become mandatory	*Obviously, we would love for it to become a school mandated vaccine, but we believe that’s outside our bailiwick…* (*Clinician 5*)

### Patient–clinician interactions

Concerns regarding parental perceptions about the connection between the HPV vaccine and sexual activity featured commonly in both clinician and stakeholder interviews. Messaging around the impact of the HPV vaccine on sexual activity was the most frequently identified theme (for clinicians *n* = 11, for stakeholders *n* = 6). Interviewees often cited how parents would raise concerns that receipt of the HPV vaccine could result in early initiation of sexual activity and more frequent sexual encounters among adolescents. As one clinician expressed, “the parents have to [give] written consent in order to administer [the] HPV vaccine. And I would say the most common concern is about sexually transmitted virus [sic]. That’s why parents refuse it…” (Clinician 7). One stakeholder noted that “in my opinion, some of it is perception about, you know, you only need the vaccine if you are sexually active, and you are going to encourage sexually active behavior if you give your kid the HPV vaccine” (Stakeholder 2). Concerns that the age at which the vaccine was recommended was too young were noted by some clinicians and were related to concerns about addressing adolescent sexual activity— “Addressing the fact that you are offering a vaccine prior to sexual activity to young people starting at the age of eleven. That often takes parents aback quite a bit” (Clinician 9).

Clinicians often also described fielding concerns from parents of adolescent patients about the safety of the HPV vaccine. Safety issues primarily came up around the idea that the vaccine may cause adverse side effects with one clinician noting “I get a lot of questions about side effects, ‘I’ve heard bad things’ or ‘I’ve heard that a child has fainted’ or something like that” (Clinician 3).

### Influence of external messaging

Discussing vaccines in the context of cancer prevention was commonly cited as a messaging tool for promotion of the HPV vaccine (Table 4). Clinicians expressed how a focus on the benefits of cancer prevention from the HPV vaccine could turn the focus of patients’ parents away from concerns about sexual activity. “I think the concern about cancer and transmission rings home” (Clinician 7). This was the most utilized method of messaging strategy among all clinicians interviewed (*n* = 11) and a primary health outcome of HPV vaccination programs identified by stakeholders (*n* = 13).

Concerns surrounding misinformation being spread on social media were also very common. Clinicians described encounters with parents who found misinformation online and how challenging they found addressing misinformation about the vaccine could be. One clinician noted that misinformation derived from the internet could raise “emotional kinds of concerns that are really hyped up … It’s pretty intense. If you bought into it, you’d but into it hook, line, and sinker. It’s got a lot of fear-based drive to it” (Clinician 10). Stakeholders shared in this frustration but highlighted that some media campaigns to raise awareness have had positive results. As one stakeholder noted, “one way to engage in this information sharing is through social media usage, with newspaper articles, mainstream media, and credible news sources to leverage this information and make vaccination the norm” (Stakeholder 9).

Bringing in outside experiences during clinician–patient interactions was frequently noted as a method to help normalize the HPV vaccine and its benefits and counter misinformation derived from other sources. Some providers shared their positive personal experiences with the vaccine as one clinician noted that “I typically share that my children have gotten or will get it and that it’s our only vaccine that prevents cancer” (Clinician 10). Most clinicians noted the impact of making a strong recommendation to get the HPV vaccine as a priority to increase its uptake. As one clinician stated, “I think having a conversation with the families to make sure they understand why we’re doing it and the importance of it—the clinician-to-family conversation. We also present it as part of the recommended immunization series, but to use, it’s the same … It’s an important vaccine that we highly recommend” (Clinician 8).

### Healthcare system-related factors

Stakeholders were asked about HPV vaccination at the organizational level. For most organizations, HPV vaccination had become more of a priority recently. However, it was often in competition with other organizational commitments: “Well, if I could say on a scale of 1 to 10, it’s not a 0 and it’s not a 10 … maybe an 8 because it is publicly reported” (Stakeholder 2).

Less than half of the clinicians we interviewed knew of ongoing quality improvement initiatives to increase HPV vaccine uptake within their organizations. Clinicians who noted quality improvement initiatives frequently cited local access expansion and clinic workflow measures as targets for increasing HPV vaccine uptake. Expanding access to the vaccine by offering it at venues outside the clinic was one commonly cited measure with a clinician noting “We also have started offering the HPV vaccine in our express clinics. We have two express clinics in the community where … they’re located at supermarkets” (Clinician 5). Changes addressing vaccines during the check-in process and leveraging the electronic medical record (EMR) system were also commonly employed to try to increase HPV vaccine uptake. One example of this was a clinician who noted that checking for vaccine receipt occurred at the time of any visit even outside of usual annual physicals—“If they’re in for any other visit outside of a yearly physical, then the rooming process includes checking the vaccine status for anybody even if it’s not for their annual visit …” (Clinician 10).

Partnerships precipitated by stakeholder groups with clinicians were felt to be crucial at improving HPV vaccine uptake. One stakeholder noted that “we have completed two rounds of a quality improvement initiative working with physicians not only in Minnesota but also in a five-state area to improve administration of first-dose HPV before age 13 for both boys and girls in their practice. We’ve seen those rates increase from 30 up to 70% as part of the second round of the quality improvement that we have done” (Stakeholder 6). For organizations in which stakeholders described an ongoing quality improvement initiative, the use of financial incentives for patients who have received the HPV vaccine was considered to result in high rates of success. One stakeholder noted that “we have member incentives in the form of [gift cards] that we are giving out to members when they get their full series of HPV vaccination” (Stakeholder 8).

## Discussion

Our study identified themes around messaging and interventions involving HPV vaccine uptake commonly encountered by clinicians and healthcare-related stakeholders practicing in Minnesota. The most commonly encountered concern raised by adolescents’ parents about the HPV vaccine during clinic visits involved its impact on sexual activity. The messaging strategy most consistently recommended among interviewees was the promotion of the HPV vaccine as a cancer prevention vaccine. Several clinicians in our study described the benefit of framing the vaccine solely in terms of its cancer prevention benefit. This insight is prescient given that efforts in other states to evaluate barriers to HPV vaccine uptake have shown that a lack of understanding about the link between cancer and the HPV vaccine is common among parents of adolescents [9, 10]. The lack of prioritization of the HPV vaccine among healthcare systems and other stakeholders within Minnesota was another important finding from this study as many clinicians noted that they were not involved with any direct quality improvement projects focused on raising HPV vaccine uptake rates in their clinics. However, there seemed to be increasing awareness of the importance of HPV vaccine administration, particularly as measures are being taken to publicly report this data in Minnesota. Increasing awareness of the benefits of increasing HPV vaccine uptake among healthcare organizations operating within Minnesota would be a good starting point for improving HPV vaccine uptake rates.

Additionally, concerns about misinformation related to the impact of social media were common among both clinician and stakeholder interviewees. Also mentioned were quality improvement initiatives and media campaigns that utilize electronic media to counter misinformation and promote the benefits of the HPV vaccine. There is an emerging literature around the possibility of utilizing social media to better understand concerns related to the HPV vaccine [11]. Some studies have analyzed how the content of messages posted on social media platforms drive varying levels of engagement and the routes through which they link to sites providing information about the HPV vaccine [11–13]. A method to leverage social media platforms to directly reach target populations who would benefit from receipt of the HPV vaccine has also been examined [14]. Specific quality improvement projects that incorporate electronic medical records and other electronic or social media were mentioned by several clinicians and stakeholders as a means to improve HPV vaccine uptake rates. Our study has helped to highlight these for future interventions that could be deployed to improve HPV vaccine uptake rates in Minnesota and elsewhere.

Our sampling strategy for identifying interviewees is a potential limitation of this study given that many of our interviewees were drawn from Minnesota’s largest metropolitan areas, potentially decreasing the viewpoints of residents of more rural parts of the state. However, we worked in conjunction with the Minnesota Department of Health to identify areas of lower vaccine uptake within Minnesota and did specific outreach in those counties of the state found to have HPV vaccine uptake rates that were below the statewide average. An additional limitation of our study was its small number of interviewees, which could reduce the perceived number of factors identified in our study as being significant to a larger group of clinicians or stakeholders (e.g., vaccine perception among specific population subgroups). However, our sampling methods allowed for outreach to interviewees over a broad geographic area within Minnesota, resulting in capture of a diverse breadth of attitudes around HPV vaccination. This can help us to build upon prior studies on this topic in Minnesota that utilized a narrower scope of recruitment [15, 16]. The results will allow for the design of more targeted qualitative and quantitative studies to evaluate factors that may be more pertinent to our state, such as the effect of including HPV vaccination as a community measure.

## Conclusion

In conclusion, clinicians and stakeholders working within Minnesota highlighted consistent messages expressed by patients and their family members that were often grounded in a lack of knowledge about the HPV vaccine or misinformation. These factors could contribute to hesitancy to receive the HPV vaccine and account for issues with increasing HPV vaccine uptake to the Healthy People 2020 target of 80% completion of the vaccine series among adolescents.

Messaging methods to counter misinformation were also described as were techniques involving quality improvement projects being implemented in some clinics as previously described. These included providing a strong provider recommendation to receive the HPV vaccine, focusing on cancer prevention benefits of the vaccine, normalizing the vaccine as part of a standard set of immunizations, leveraging clinic workflow and EMR systems, using social media campaigns to promote awareness and factual information about the vaccine, and utilizing novel strategies such as financial incentives to promote vaccine uptake. The information gleaned from this study will be informative for designing clinical interventions at a larger scale to help increase HPV vaccine uptake. While this study was conducted within Minnesota, the lessons learned can be applied more generally to other communities outside the state.

## Data Availability

Transcribed interviews are maintained electronically at the University of Minnesota and are available for viewing upon request.
